# Supplementary material for: Two-dimensional amorphous NiO as a plasmonic photocatalyst for solar H2 evolution
Source: Nat Commun. 2018 Oct 2;9:4036. doi: 10.1038/s41467-018-06456-y (PMC6168506; doi:10.1038/s41467-018-06456-y)
Supplement: Supplementary file 1 — Supplementary Information [file 41467_2018_6456_MOESM1_ESM.pdf]

Supplementary Information

Two-dimensional amorphous NiO as a plasmonic photocatalyst for solar H_2 evolution

Lin et al.

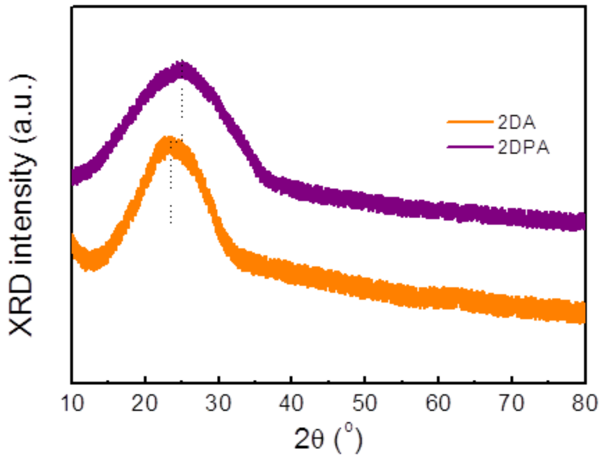

Supplementary Fig. 1 XRD patterns of 2DA and 2DPA.

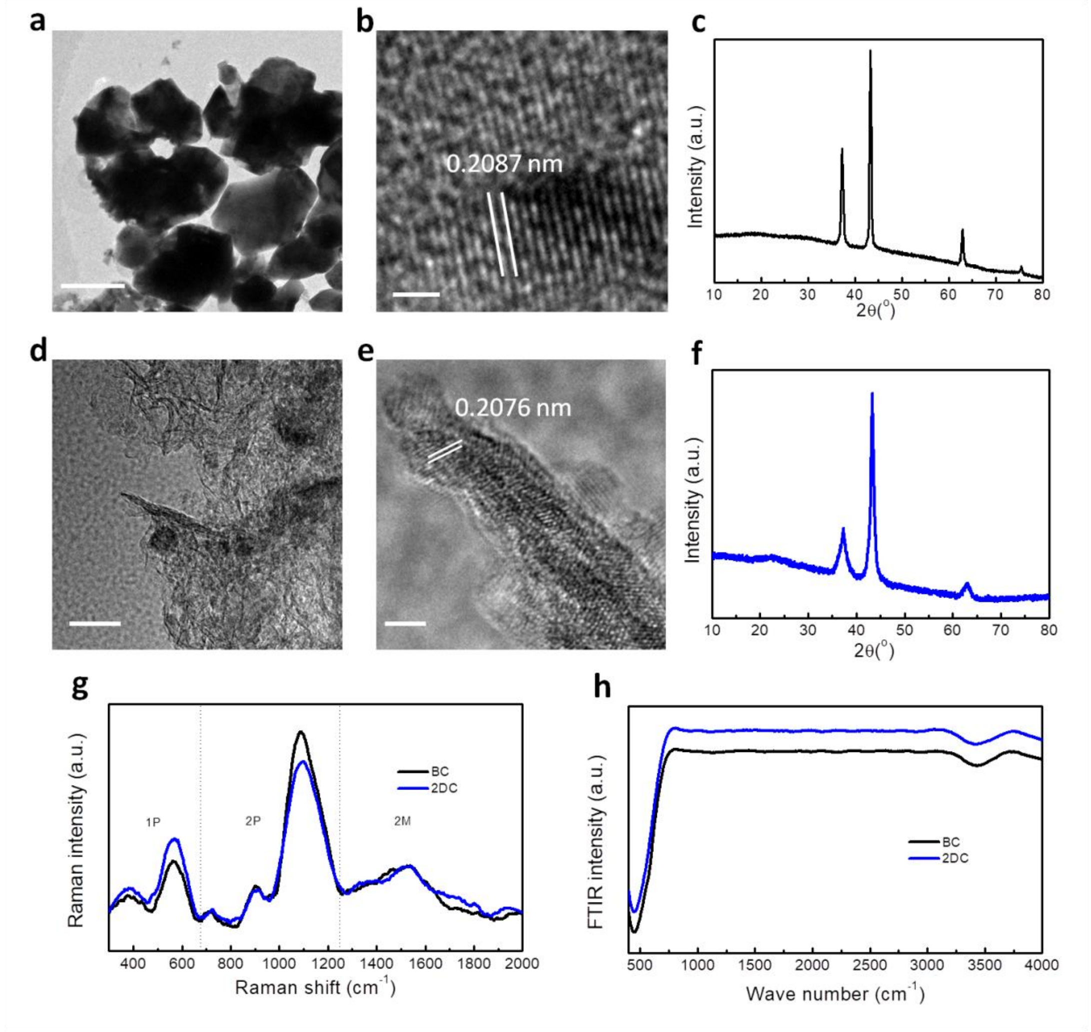

Supplementary Fig. 2 Morphologies and structures of BC and 2DC. **a-c** TEM (**a**), HRTEM (**b**) images and XRD pattern (**c**) of BC. **d-f** TEM (**d**), HRTEM (**e**) images and XRD pattern (**f**) of 2DC. **g-h** Raman (**g**) and FTIR (**h**) spectra of BC (black) and 2DC (blue). Scale bars, 500, 1, 200 and 2 nm in **a**, **b**, **d** and **e**, respectively.

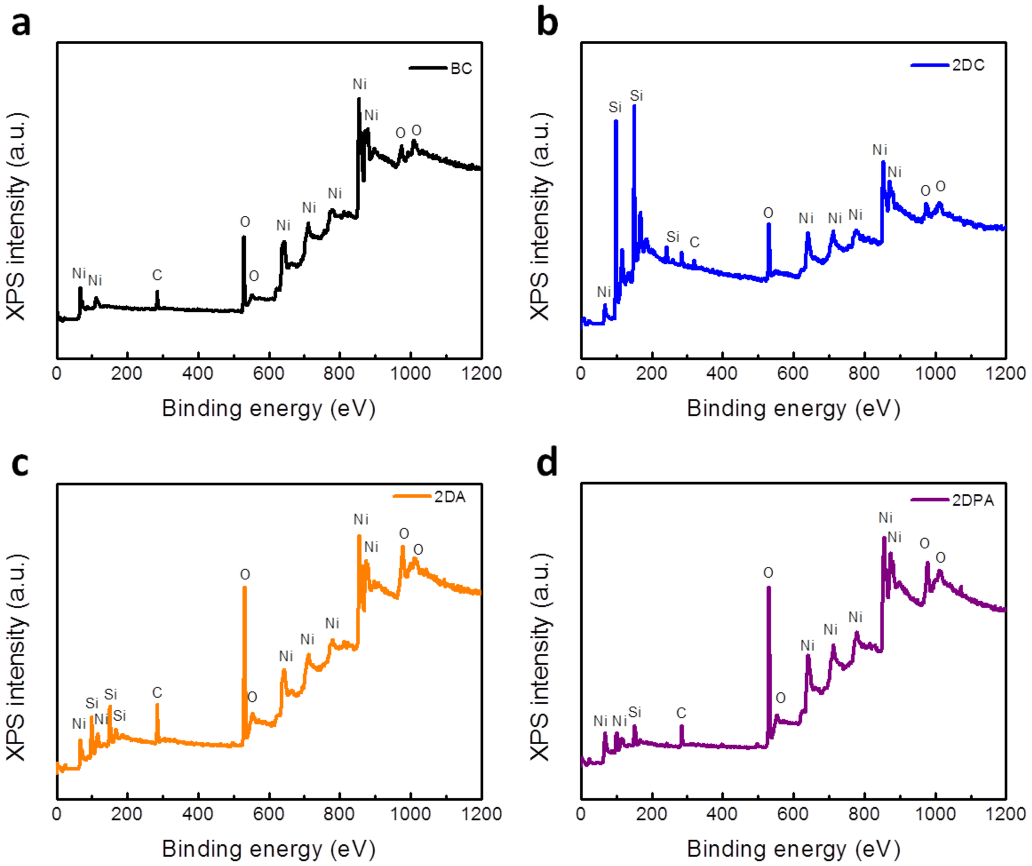

Supplementary Fig. 3 XPS spectra of the four samples. **a** BC, **b** 2DC, **c** 2DA and **d** 2DPA.

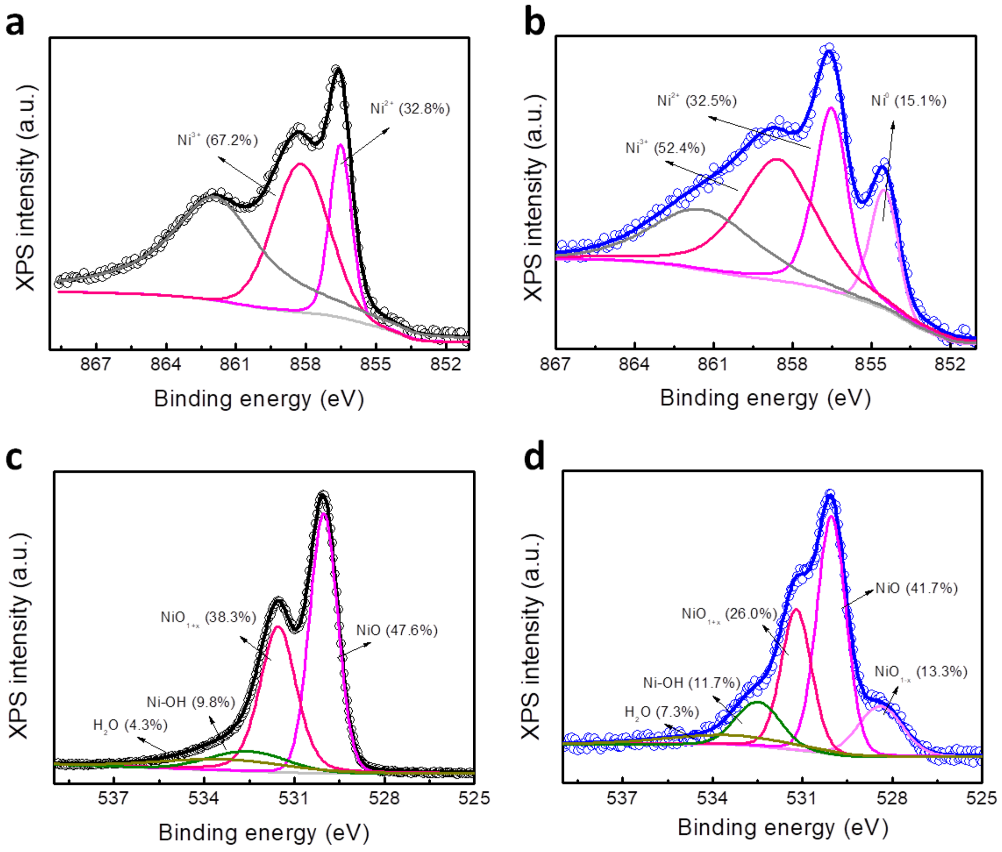

Supplementary Fig. 4 Ni $2p_{3/2}$ and O 1s XPS spectra of BC (black) and 2DC (blue).

a, b Ni $2p_{3/2}$ XPS spectra, **c, d** O 1s XPS spectra.

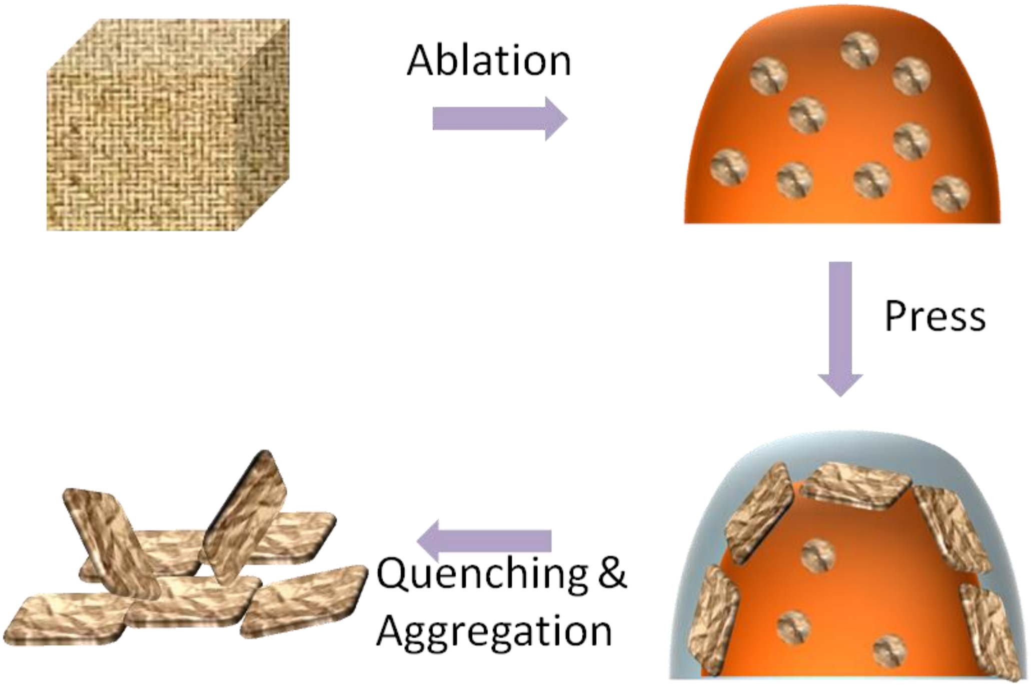

Supplementary Fig. 5 The growth process of amorphous nanoflakes involving laser ablation in liquids.

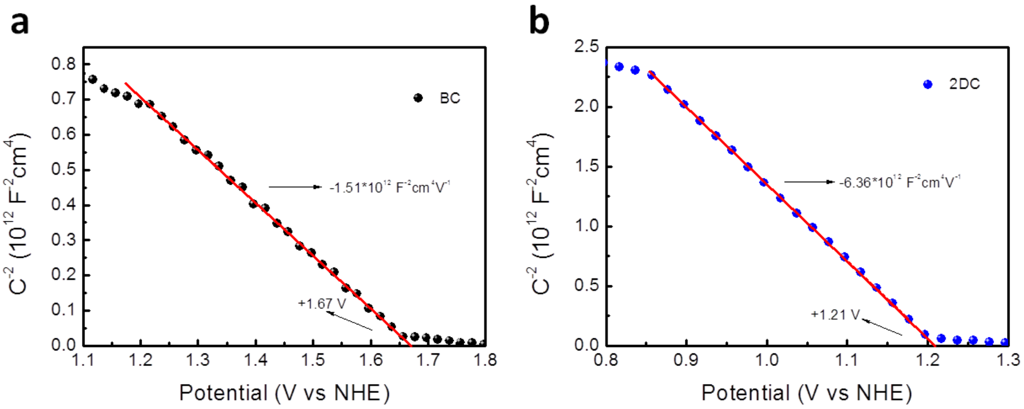

Supplementary Fig. 6 Mott-Schottky plots of a BC and b 2DC.

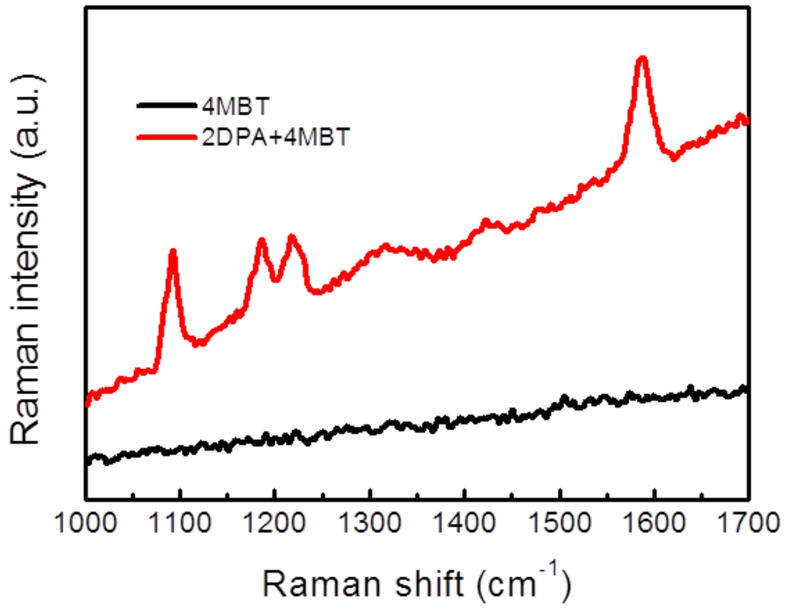

Supplementary Fig. 7 Raman spectra of 4-MBT in the absence (black) and in the presence of 2DPA (red).

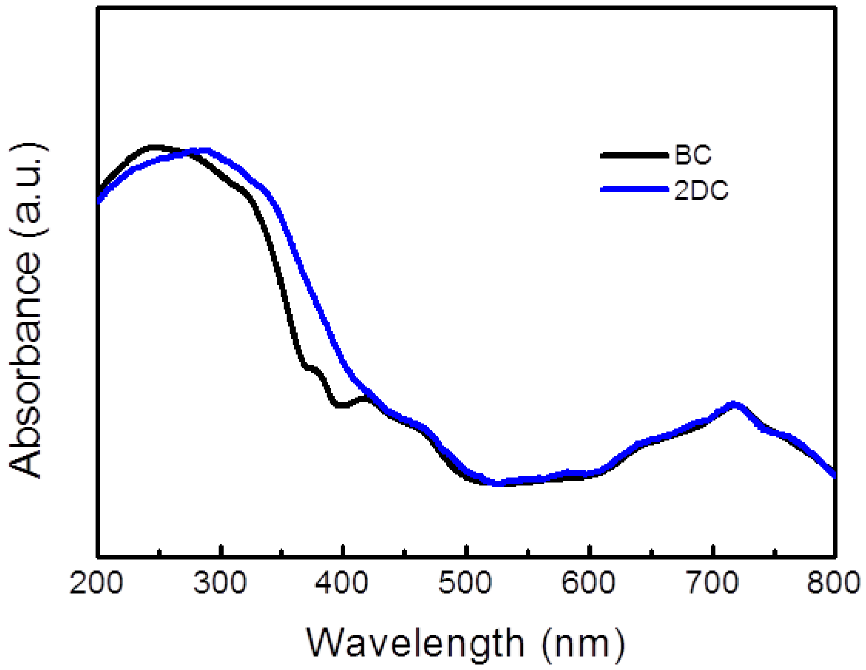

Supplementary Fig. 8 Absorbance spectra of BC (black) and 2DC (blue).

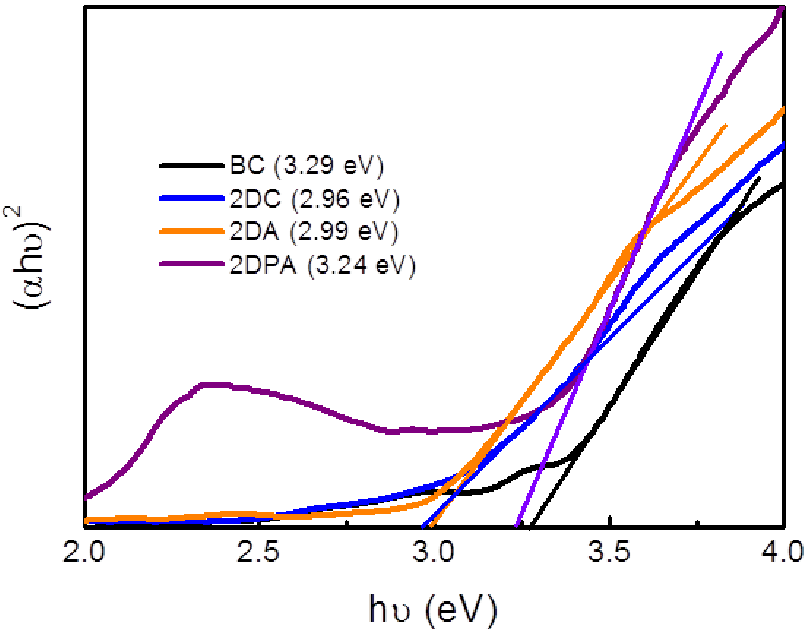

Supplementary Fig. 9 Tauc plots of BC (black), 2DC (blue), 2DA (orange) and 2DPA (purple).

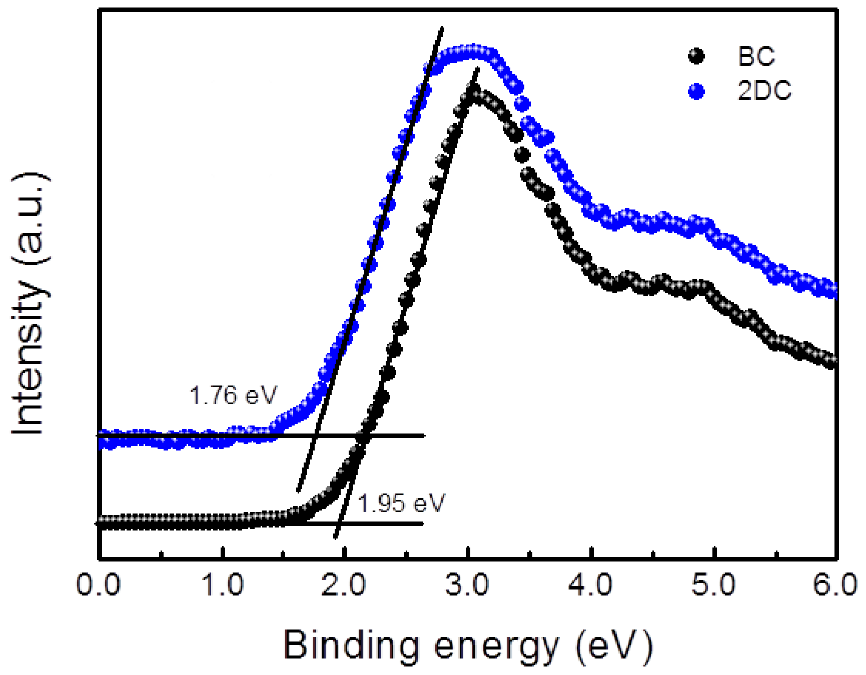

Supplementary Fig. 10 VB XPS spectra of BC (black) and 2DC (blue).

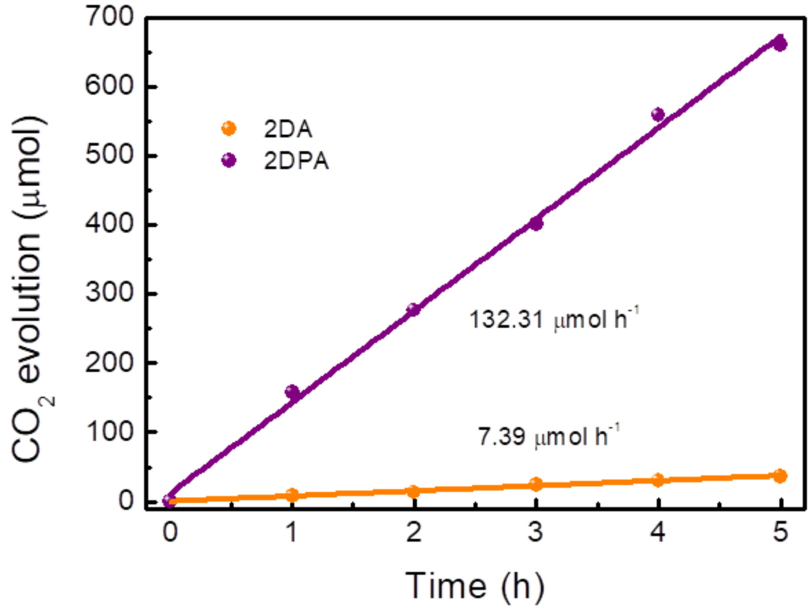

Supplementary Fig. 11 Typical time courses of CO_2 evolution upon AM 1.5 over 2DA and 2DPA in 20 vol.% methanol solution.

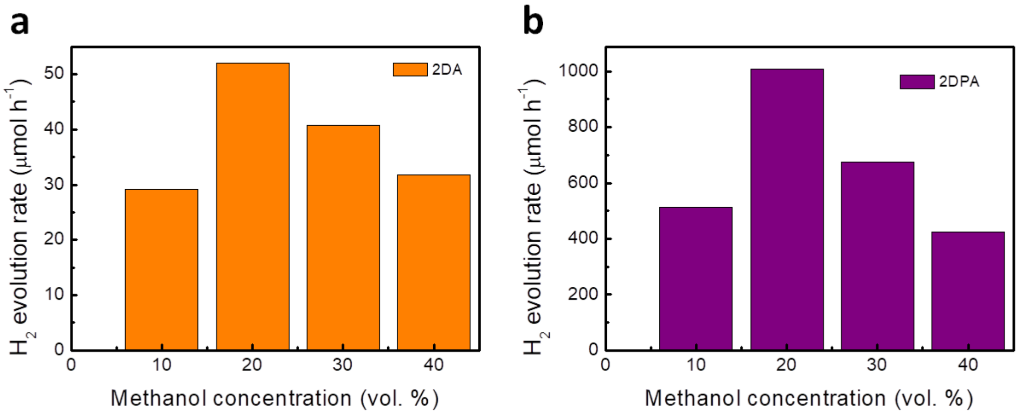

Supplementary Fig. 12 H_2 evolution rate vs. methanol concentration upon AM 1.5 irradiation over the two amorphous materials. **a** 2DA and **b** 2DPA.

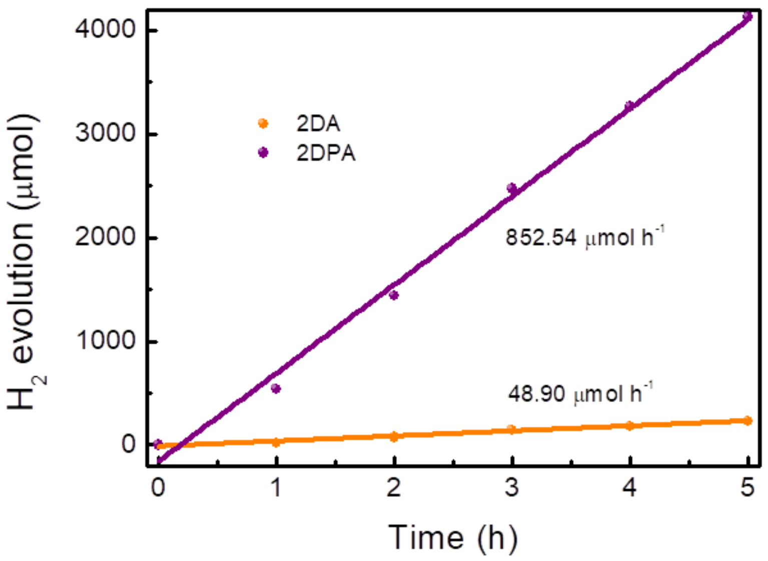

Supplementary Fig. 13 Typical time courses of H_2 evolution upon AM 1.5 over 2DA and 2DPA in Na_2S/Na_2SO_3 aqueous solution.

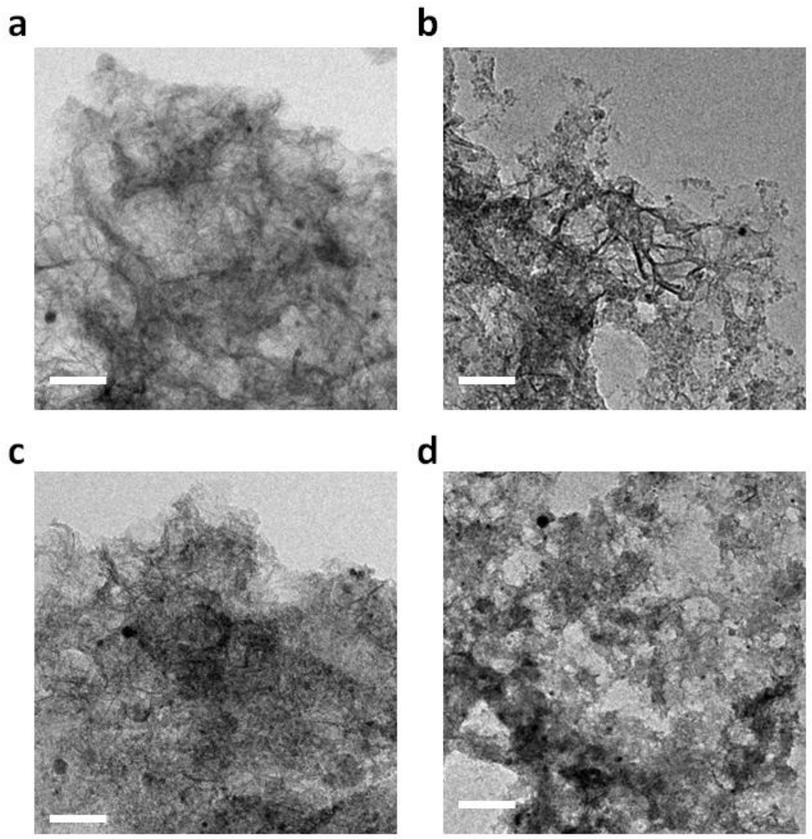

Supplementary Fig. 14 TEM images of the samples for photocatalytic oxygen evolution tests. **a** 2DA-A, **b** 2DPA-A, **c** 2DA-B and **d** 2DPA-B. Scale bars, 50 nm.

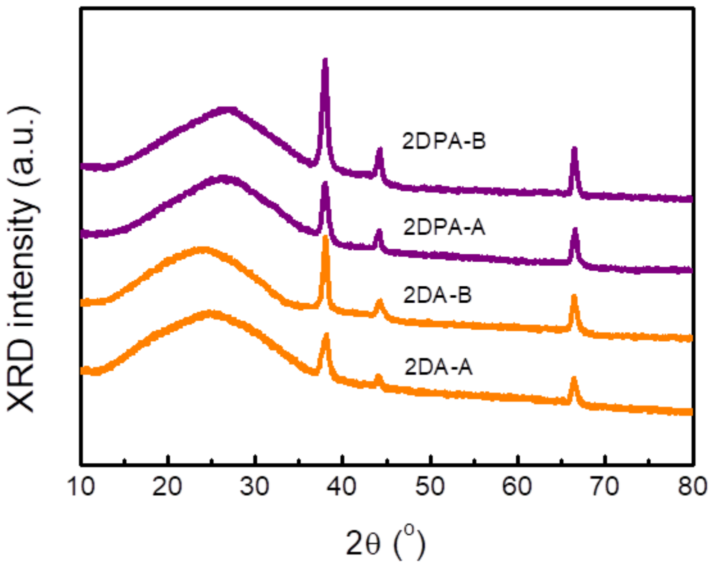

Supplementary Fig. 15 XRD patterns of 2DA-A, 2DA-B, 2DPA-A and 2DPA-B.

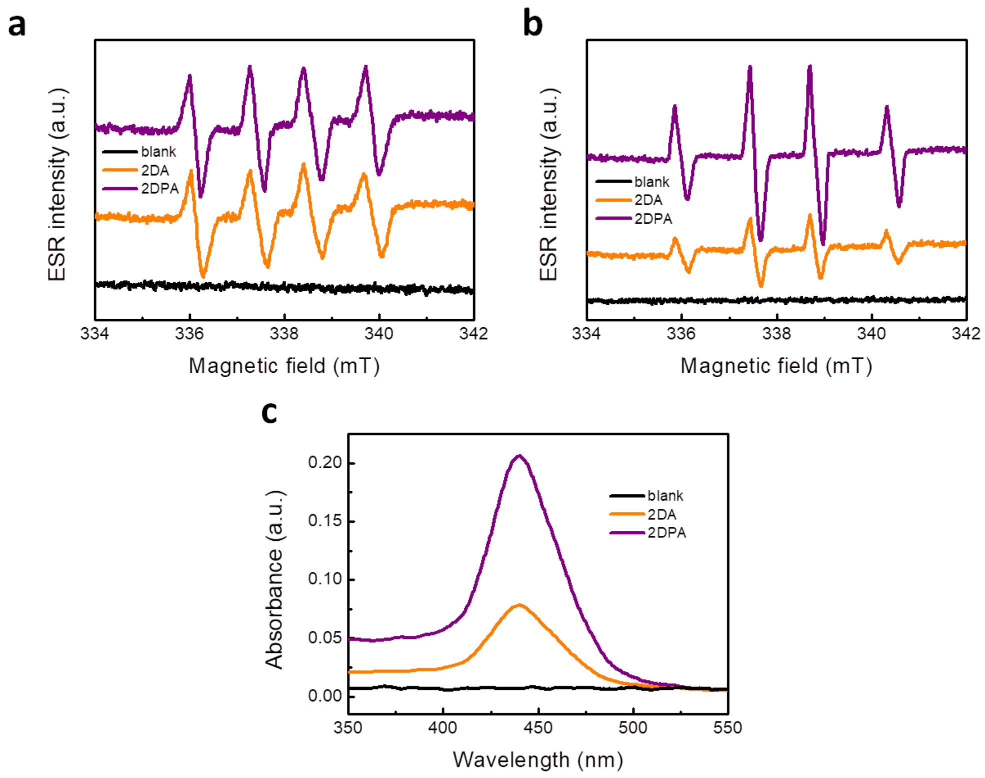

Supplementary Fig. 16 ROS detection for the $AgNO_3$ solution upon AM 1.5 irradiation. ESR spectra of **a** the BMPO-$\cdot O_2^-$ adducts, **b** the DMPO-·OH adducts over 2DA and 2DPA. **c** Absorbance spectra of the filtrate after photocatalytic tests for H_2O_2 detection.

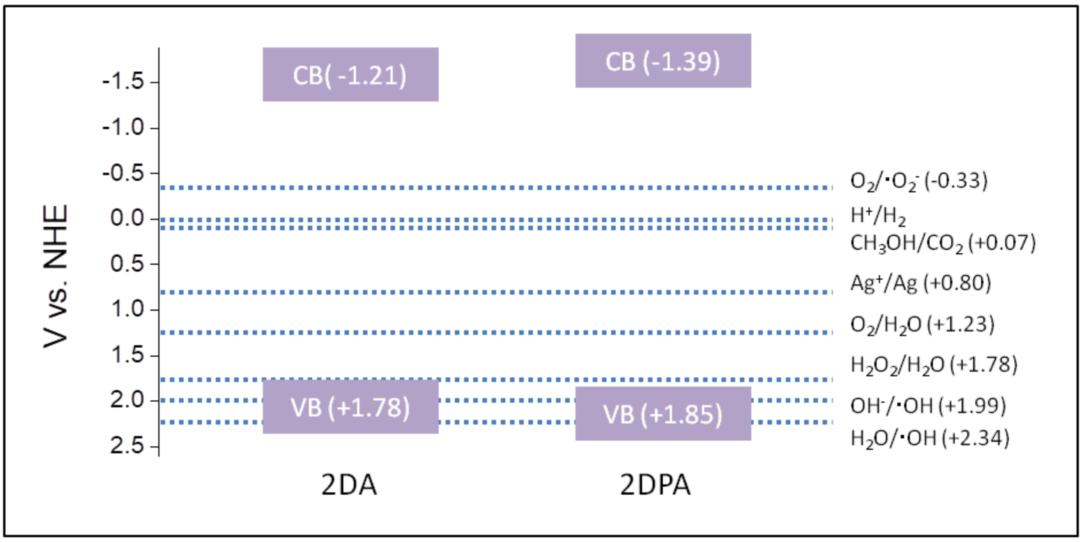

Supplementary Fig. 17 Schematic representation of the band structures and the involved redox potentials.

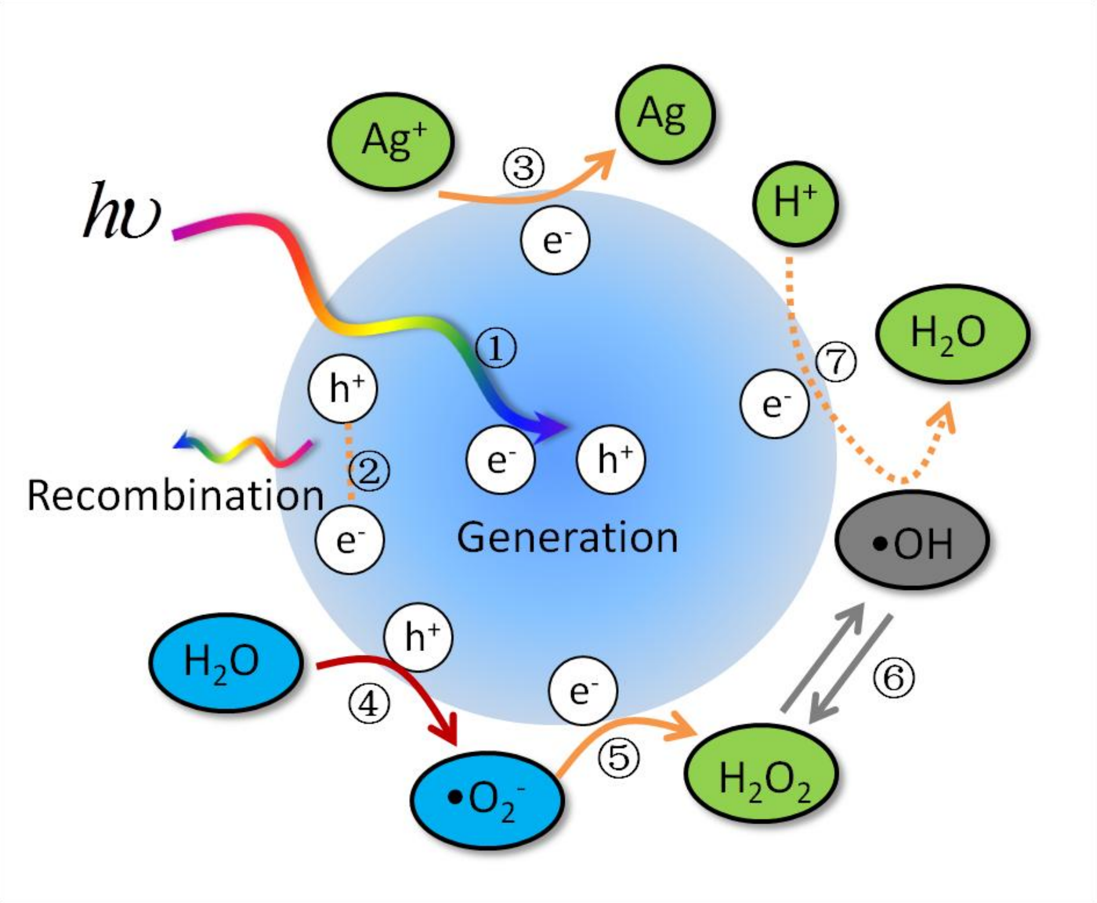

Supplementary Fig. 18 The proposed possible ROS evolution route for the photocatalytic tests in $AgNO_3$ solution.

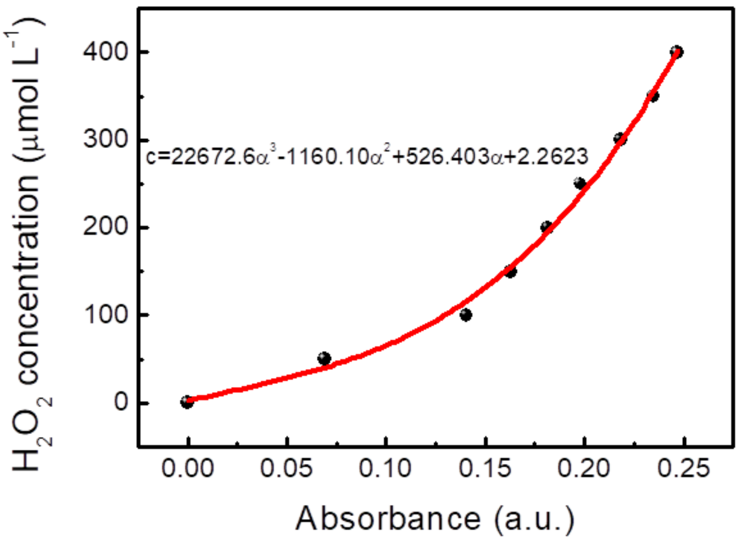

Supplementary Fig. 19 The calibration curve between H_2O_2 concentration and absorbance of the coloration solution. α and c are respectively the absorbance and the H_2O_2 concentration. A three-order polynomial was used for fitting.

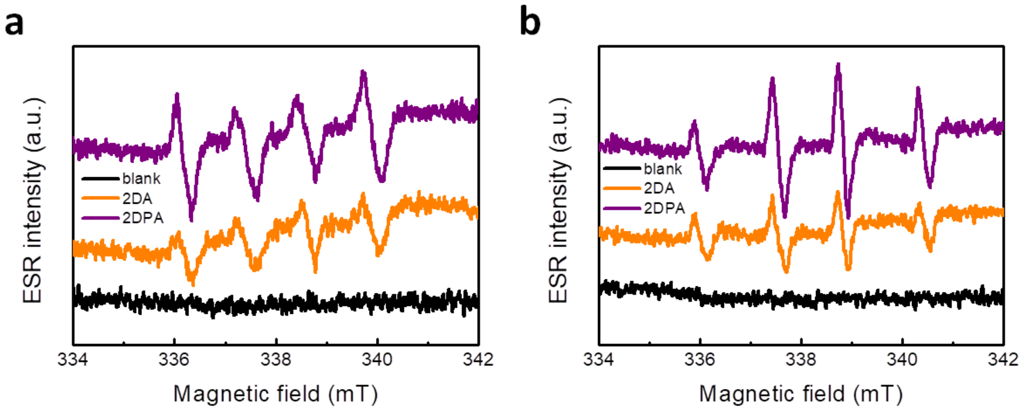

Supplementary Fig. 20 ROS detection for pure water upon AM 1.5 irradiation. ESR spectra of **a** the BMPO-$\cdot O_2^-$ adducts, **b** the DMPO-$\cdot$OH adducts over 2DA and 2DPA.

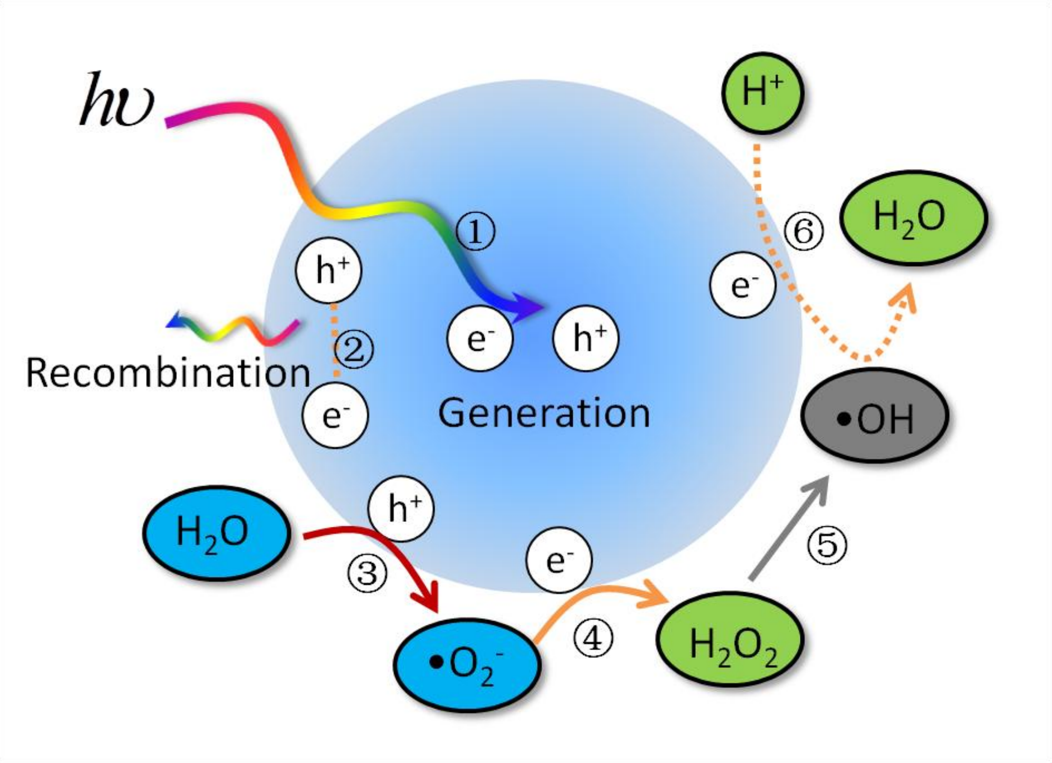

Supplementary Fig. 21 The proposed possible ROS evolution route for the photocatalytic tests in pure water.

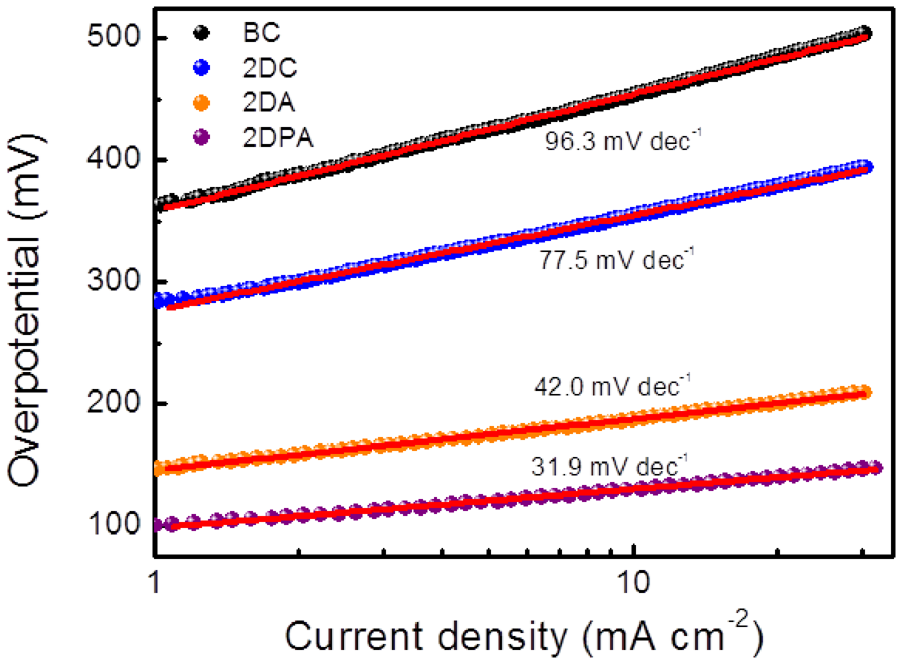

Supplementary Fig. 22 Tafel plots of BC, 2DC, 2DA and 2DPA.

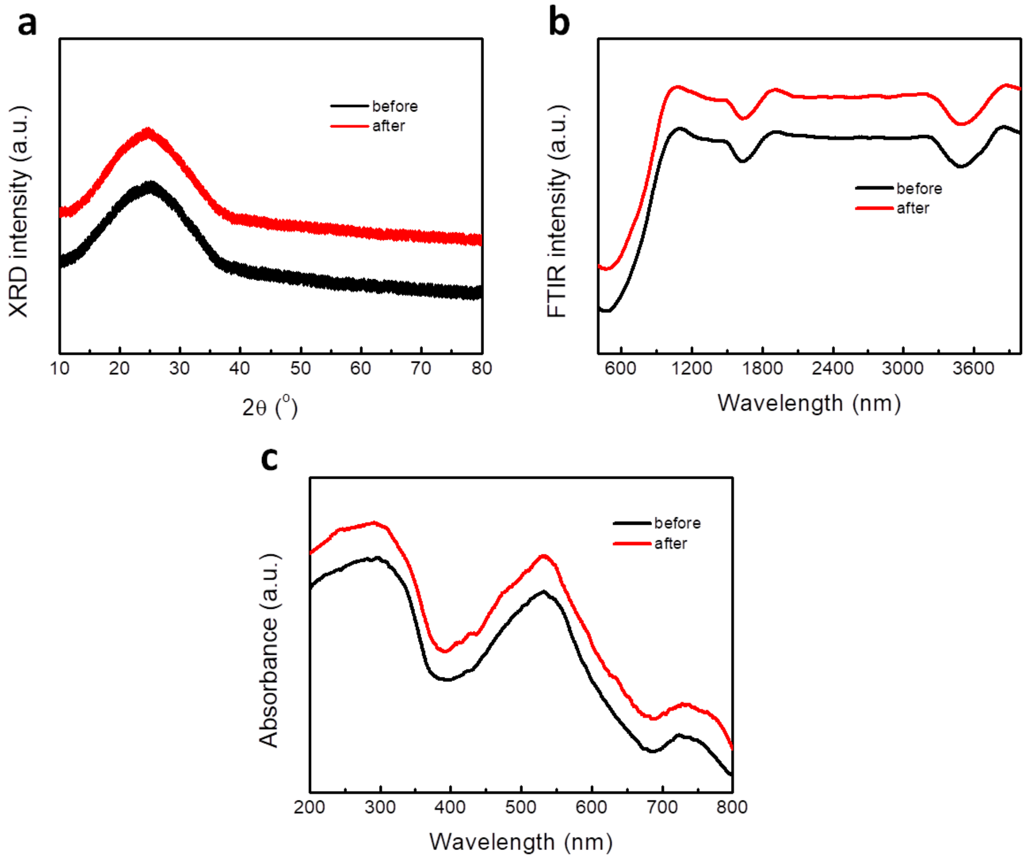

Supplementary Fig. 23 Comparison of the 2DPA samples before and after the long-term test. **a** XRD patterns, **b** FTIR spectra and **c** absorbance spectra.

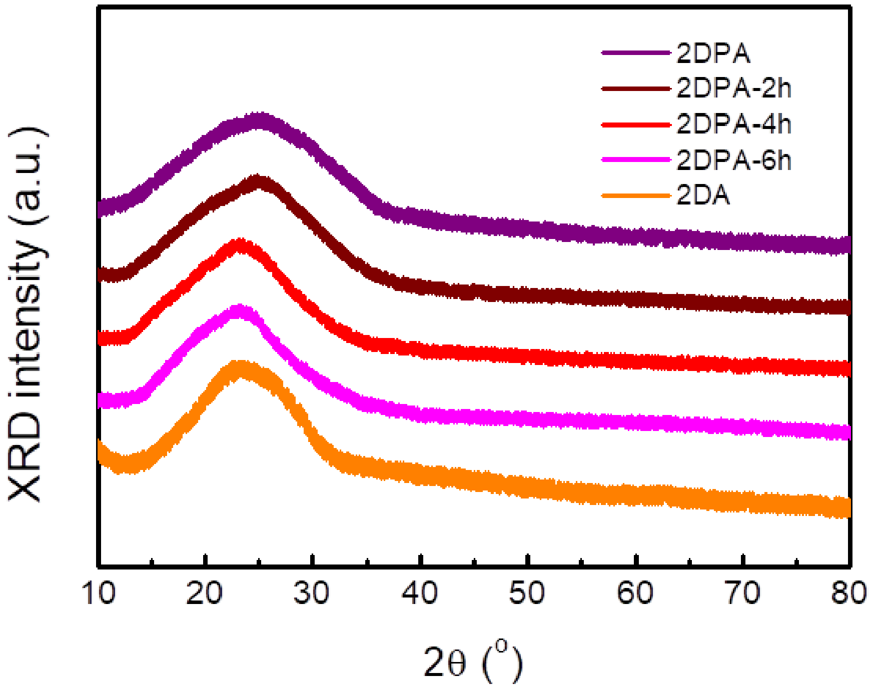

Supplementary Fig. 24 XRD patterns of 2D amorphous NiO nanoflakes with various H doping amounts.

Supplementary Table 1 Deconvolution of the Ni $2p_{3/2}$ XPS spectra.

**Sample**	**Ni^{2+} (%)**	**Ni^{3+} (%)**	**Ni^{0}-like defects (%)**
**BC**	32.8	67.2	—
**2DC**	32.5	52.4	15.1
**2DA**	64.9	—	35.1
**2DPA**	53.6	—	46.4

Supplementary Table 2 Deconvolution of the O 1s XPS spectra.

**Sample**	**NiO_{1-x} (%)**	**NiO (%)**	**NiO_{1+x} (%)**	**Ni-OH (%)**	**H_2O (%)**
**BC**	—	47.6	38.3	9.8	4.3
**2DC**	13.3	41.7	26.0	11.7	7.3
**2DA**	18.5	41.8	—	17.4	22.2
**2DPA**	28.1	35.8	—	16.4	19.7

Supplementary Note 1

As shwon in Supplementary Fig. 2a, it can be clearly seen that BC is irregular with a size of several hundred nanometers. HRTEM image in Supplementary Fig. 2b presents a typical interplanar spacing of 0.2087 nm, which corresponds to the (200) crystallographic plane of cubic bunsenite NiO phase (JCPDS 47-1049). 2DC is in the shape of nanoflake with a thickness less than 10 nm, similar to 2DA and 2DPA (Supplementary Fig. 2d). The (200) crystallographic plane of cubic NiO phase with a interplanar spacing of 0.2076 nm demonstrates the crystallization (Supplementary Fig. 2e). The corresponding XRD patterns are shown in Supplementary Fig. 2c and 2f. The sharp and narrow diffraction peak at 37.2^o, 43.3^o and 62.9^o can be index to (111), (200) and (220) planes of cubic bunsenite NiO (JCPDS 47-1049), respectively. It indicates that both BC and 2DC are crystalline. Compared with the XRD pattern of BC, we can clearly find that the peaks of 2DA are widened. It would be due to the reduced size.

Raman spectra of BC and 2DC are shown in Supplementary Fig. 2g. The measurements were carried out at room temperature using argon ion laser (514.5 nm, 90 mW). The Raman spectrum shows five distinguishable bands. The first four ones are vibration bands. The bands at 382.4 and 565.7 cm^{-1} can be assigned to the first-phonon modes (1P). They are first-order transverse optical (TO) and longitudinal optical phonon modes (LO), respectively. The bands at 713.1, 903.8 and 1087.1 cm^{-1} can be assigned to the two-phonon (2P) modes. They are two-order transverse optical (2TO), transverse optical+longitudinal optical (TO+LO), two-order longitudinal

optical phonon modes (2LO), respectively. The last band at 1532.6 cm^{-1} can be assigned to the two-magnon scattering mode (2M). 1P modes of 2DC are slightly weaker than those of BC, suggesting 2DC is slightly disordered due to the 2D effect.

As shown in the FTIR spectra in Supplementary Fig. 2h, the band near 450 cm^{-1} is attributed to the stretching vibration mode of the Ni-O bond. The band near 3400 cm^{-1} is ascribed to the adsorbed H_2O. Compared with 2DA and 2DPA, BC and 2DC possess narrower Ni-O bands, indicating their high crystallinity.

Supplementary Note 2

As shown in the Ni $2p_{3/2}$ XPS spectra in Supplementary Fig. 4a and b, the peaks plotted in grey at about 861.9 eV are assigned to Ni $2p_{3/2}$ satellite peaks due to the existence of Ni^{2+}. The satellite peaks are decreasing in the order of BC, 2DC, 2DA, 2DPA, suggesting the increasing doping degree. The peaks at 854.5, 856.5 and 858.6 eV are attributed to Ni^0-like defects, Ni^{2+} and Ni^{3+}, respectively.

As shown in the O 1s XPS spectra in Supplementary Fig. 4c and d, the peaks at binding energies of 528.4, 530.1, 531.3, 532.6 and 534.1 eV are respectively assigned to oxygens in under-coordinated nickel oxides (NiO_{1-x}), stoichiometric nickel oxides (NiO), over-coordinated nickel oxides (NiO_{1+x}), nickel-hydroxyl groups (Ni-OH) on the surface and adsorbed H_2O.

Supplementary Note 3

BC powders were dispersed in water, and ablated by high-energy laser. Ni-O bonds are broken first by laser ablation. A plasma plume was generated on the interface between the solid NiO and the confining water, which contained numerous neutral atoms, ions and electrons from the NiO powders. The temperature and pressure inside are usually very large. This situation is far away from the thermodynamic equilibrium, and suitable for the production of meta-stable materials.

The plasma plume was inclined to expand, however, the expansion was strongly confined by water. A shock wave was then induced by the continual supply of vaporizing species in the plasma plume. This shock wave resulted in extra pressure in the plasma plume, called plasma-induced pressure. During the expansion and condensation of the plasma plume, energy was transferred to the surrounding water. Therefore, a thin layer of vapor appeared and evolved to a cavitation bubble around the plasma plume. This cavitation bubble produced an additional pressure over the plasma plume. It pressed the plasma back against the ablation spot. This is the so-called pressing process. Thanks to the large specific heat capacity of water, the plasma plume quenches quickly during the intermission of laser pulses. Since the quenching time was extremely short (about tens of picoseconds), the disordered distribution of Ni and O species in the plasma plume was reserved in the product released from the plasma plume. The morphology of the product was flake-like due to the pressing process. Finally, the amorphous nanoflakes aggregated due to the large surface areas of the nanoflakes.

It is believed that a water environment is necessary for the formation of amorphous phase by laser ablation on account of its large specific heat capacity for the rapid cooling process[1,2]. In our case, 2DPA is prepared in methanol-water mixture with a smaller specific heat capacity. Surprisingly, 2DPA is more disordered than 2DA, demonstrated by the TEM, XRD and Raman results. It should be because of the formation of Ni-H bonds (FTIR and ^{1}H NMR spectra). It is in accord with our previous work revealing a reducing environment, in this case, methanol, is necessary for the H doping[3].

Supplementary Note 4

Since crystalline NiO is a direct band gap semiconductor, the optical band gap is estimated using the Tauc plot of $(\alpha \mathrm{h}\upsilon)^2$ *vs.* $\mathrm{h}\upsilon$. α, h and υ are the absorbance, Plank constant and light frequency, respectively. Therefore, the optical band gaps of BC, 2DC, 2DA and 2DPA were respectively estimated to be 3.29, 2.96, 2.99 and 3.24 eV by linear extrapolation of the leading edges to the base lines.

Supplementary Note 5

An isotope labelling study is generally employed to determine the origin of the evolved gases in photocatalytic overall water splitting[4,5]. However, it may be not applicable in our case. As mentioned by Kandiel, H^+ is easier to be reduced than D^+. An isotope labelling will influence the reaction pathways to some extent[6]. Further, the rapid and frequent H^+/D^+ isotopes exchange will take place in the whole photocatalytic process, making it difficult to determine the origin.

Supplementary Note 6

As shown in Supplementary Fig. 16a, in the absence of a photocatalyst, no signal could be detected (labelled as blank). When photocatalyst powders were added into the system, a four-line spectrum with the relative intensities of 1:1:1:1 formed, which is the characteristic spectrum for the BMPO-$\cdot O_2^-$ adducts[7]. Similarly, four characteristic peaks with the intensity ratios of 1:2:2:1 appeared when DMPO was used as the trapping agent, shown in Supplementary Fig. 16b. These peaks should be attributed to the DMPO-$\cdot$OH adducts[8]. On the contrary, no TEMP-$^1O_2$ adducts was detected (triplet signals), indicating the absence of $^1O_2$ radicals in the process[9]. The generation of H_2O_2 was determined by the coloration method, and the absorbance spectra are shown in Supplementary Fig. 16c. The spectra show a characteristic peak with the wavelength of 439 nm, demonstrating the existence of H_2O_2 in the reaction solutions[10]. Evidently, $\cdot O_2^-$ radicals, H_2O_2 and $\cdot$OH radicals can be detected in the $AgNO_3$ solution containing 2DA or 2DPA upon irradiation, whereas $^1O_2$ was absent. Compared with 2DA, 2DPA owns stronger signals for the detection of $\cdot O_2^-$, H_2O_2 and $\cdot$OH, which should be due to its stronger light harvesting ability.

Supplementary Note 7

According to the ROS detection results, it is found that $\cdot O_2^-$, H_2O_2 and $\cdot OH$ are generated in the $AgNO_3$ solution upon irradiation. Considering that the oxidation potentials of H_2O to $\cdot OH$ (+2.34 V *vs.* NHE) and OH^- to $\cdot OH$ (+1.99 V *vs.* NHE) are more positive than the VB tops of 2DA (+1.78 V *vs.* NHE) and 2DPA (+1.85 V *vs.* NHE)[11], we believe that $\cdot OH$ radicals should not be generated through the direct oxidation of H_2O or OH^-. Namely, $\cdot OH$ should not be produced first. Two routes for the ROS evolution may be involved (Route A: $H_2O \rightarrow \cdot O_2^- \rightarrow H_2O_2 \rightarrow \cdot OH$; Route B: $H_2O \rightarrow H_2O_2 \rightarrow \cdot O_2^-/\cdot OH$), which can be described by the following equations.

Route A[5,12]:

$$2H_2O + 3h^+ \rightarrow \cdot O_2^- + 4H^+ \quad (1)$$

$$\cdot O_2^- + 2H^+ + e^- \rightarrow H_2O_2 \quad (2)$$

$$H_2O_2 \rightarrow 2\cdot OH \quad (3)$$

Route B[13,14]:

$$2H_2O + 2h^+ \rightarrow H_2O_2 + 2H^+ \quad (4)$$

$$H_2O_2 + h^+ \rightarrow \cdot O_2^- + 2H^+ \quad (5)$$

$$H_2O_2 \rightarrow 2\cdot OH \quad (6)$$

The oxidation potential of H_2O to H_2O_2 is reported as +1.78 V *vs.* NHE[11]. Given that the VB tops of 2DA and 2DPA are very close to this value, we consider that Route A may be more applicable to this case, which has also been reported by Tian *et al.*[5]. Certainly, one may consider that in this process, another route (Route C: $H_2O \rightarrow O_2 \rightarrow \cdot O_2^- \rightarrow H_2O_2 \rightarrow \cdot OH$) should be involved, in which O_2 is generated first.

However, on one hand, O_2 was not detected. On the other hand, the oxidation of H_2O to O_2 is a four-electron process requiring a large overpotential and more active sites to realize O-O coupling[15,16,17]. Therefore, Route C may be less possible in our case.

The possible reaction route for the system containing $AgNO_3$ and photocatalyst (2DA or 2DPA) upon irradiation is illustrated in Supplementary Fig. 18. Upon irradiation, electrons and holes are generated (①). Parts of them are recombined unavoidably (②). Due to the strong electron-harvesting ability of Ag^+, the electrons are consumed by the Ag^+ ions quickly, and Ag nanoparticles are deposited onto the surface of the photocatalyst (③). The remaining holes oxidize H_2O to generate $\cdot O_2^-$ radicals (④). H_2O_2 is further produced through the reduction of $\cdot O_2^-$ by the electrons (⑤). Meanwhile, the decomposition of H_2O_2 and the dimerization of $\cdot OH$ proceed (⑥). The reaction between $\cdot OH$ and H^+ to form H_2O again may take place, as well (⑦). Among the ROS involved above, H_2O_2 is the most stable[18]. Therefore, the whole process can be described simply by the equation

$$2Ag^+ + 2H_2O \rightarrow 2Ag + H_2O_2 + 2H^+ \qquad (7)$$

Further, when methanol was added, no ROS can be detected. It indicates that the photo-generated holes can be consumed by methanol quickly. Recombination between electrons and holes (②) is suppressed, and more electrons are left for the Ag^+ reduction (③). Therefore, more Ag nanoparticles are formed.

Supplementary Note 8

Ag^+ is a well-known electron scavenger that can consume the photo-generated electrons timely and retard the undesired carrier recombination[19]. At this point, ROS generation can proceed more smoothly. When the photocatalytic tests are conducted in pure water without Ag^+, the consumption of electrons is weakened since H^+ ions are less easily reducible than Ag^+ ions[20]. Actually, electron consumption and hole consumption are interactional. Although some of the holes can be used for producing ROS ($\cdot O_2^-$ and $\cdot OH$), they cannot be consumed quickly due to the excess of electrons. As shown in Supplementary Fig. 20, the signal to noise ratios of the ESR spectra are much smaller than those in Supplementary Fig. 16. The accumulation of electrons and holes in the photocatalysts will enhance the carrier recombination (②).

Supplementary Note 9

Generally, there are three pathways for the SPR-mediated photocatalysis based on the traditional antenna-reactor heterostructures: charge transfer, energy transfer and plasmonic heating[21]. In our case, 2DPA is a single-component photocatalyst with an incorporate antenna-reactor structure, which may obey a mechanism different from the charge transfer or energy transfer[3]. Plamonic heating is also negligible since the photocatalytic rate shows a linear correlation with the incident light intensity[22].

Supplementary References

1. Zeng, H. *et al.* ZnO-based hollow nanoparticles by selective etching: Elimination and reconstruciton of metal-semiconductor interface, improvement of blue emission and photocatalysis. *ACS Nano* **2**, 1661-1670 (2008).

2. Xiao. J., Liu, P., Wang, C. & Yang, G. External field-assisted laser ablation in liquid: An efficient strategy for nanocrystal synthesis and nanostructure assembly. *Prog. Mater. Sci.* **87**, 140-220 (2017).

3. Lin, Z., Li, W. & Yang, G. Hydrogen-interstitial $CuWO_4$ nanomesh: A single-component full spectrum-active photocatalyst for hydrogen evolution. *Appl. Catal., B* **227**, 35-43 (2018).

4. Liu, J. *et al.* Metal-free efficient photocatalyst for stable visible water splitting via a two-electron pathway. *Science* **347**, 970-974 (2015).

5. Tian, B. *et al.* Supported black phosphorus nanosheets as hydrogen-evolving photocatalyst achieving 5.4% energy conversion efficiency at 353 K. *Nat. Commun.* **9**, 1397 (2018).

6. Kandiel, T. A., Ivanova, I. & Bahnemann, D. W. Long-term investigation of the photocatalytic hydrogen produciton on platinized TiO_2: an isotopic study. *Energy Environ. Sci.* **7**, 1420-1425 (2014).

7. He, W. *et al.* Predicting and identifying reactive oxygen species and electrons for photocatalytica metal sulfide micor-nano structures. *J. Catal.* 320, 97-105 (2014).

8. Wang, Z., Ma, W., Chen, C., Ji, H. & Zhao, J. Probing paramagnetic species in titania-based heterogeneous photocatalysis by electron spin resonance (ESR) spectroscopy-A mini review. *Chem. Eng. J.* 170, 353-362 (2011).

9. Gao, B., Iftekhar, S., Srivastava, V., Doshi, G. & Sillanpää, M. Insights into the generation of reactive oxygen species (ROS) over polythiophene/$ZnIn_2S_4$ based on different modification processing. *Catal. Sci. Technol.* 8, 2186-2194 (2018).

10. Shi, W. *et al.* Carbon dots anchored on octahedral CoO as a stable visible-light-responsive composite photocatalyst for overall water splitting. *J. Mater. Chem. A* 5, 19800-19807 (2017).

11. Hisatomi, T., Kubota, J. & Domen, K. Recent advances in semiconductors for photocatalytic and photoelectrochemical water splitting. *Chem. Soc. Rev.* 43, 7520-7535 (2014).

12. Lin, Z. Y., Xiao, J., Yan, J. H., Liu, P., Li, L. H. & Yang, G. W. Ag/AgCl plasmonic cubes with ultrahigh activity as advanced visible-light photocatalysts for photodegrading dyes. *J. Mater. Chem. A* 3, 7649-7658 (2015).

13. Nosaka, Y. & Nosaka, A. Y. Generation and detection of reactive oxygen species in photocatalysis. *Chem. Rev.* 117, 11302-11336 (2017).

14. Ge, S. & Zhang, L. Efficient visible light driven photocatalytic removal of RhB and NO with low temperature synthesized $In(OH)_xS_y$ hollow nanocubes: A comparative study. *Environ. Sci. Technol.* 45, 3027-3033 (2011).

15. Liu, J. *et al.* Metal-free efficient photocatalyst for stable visible water splitting via a two-electron pathway. *Science* 347, 970-974 (2015).

16. Chen, Z. *et al.* Amorphous cobalt oxide nanoparticles as active water-oxidation catalysts. *ChemCatChem* 9, 3641-3645 (2017).

17. She, X. *et al.* High efficiency photocatalytic water splitting using 2D α-Fe_2O_3/g-C_3N_4 Z-scheme catalysts. *Adv. Energy Mater.* 7, 1700025 (2017).

18. Zhang, H., Guo, L., Zhao, L., Wan, B. & Yang, Y. Switching oxygen reduction pathway by exfoliating graphitic carbon nitride for enhanced photocatalytic phenol degradation. *J. Phys. Chem. Lett.* 6, 958-963 (2015).

19. Schnelder, J. & Bahnemann, D. W. Undesired role of sacrificial reagents in photocatalysis. *J. Phys. Chem. Lett.* 4, 3479-3483 (2013).

20. Li, X, Yu, J. Low, J. Fang, Y., Xiao, J. & Chen, X. Engineering heterogeneous semiconductors for solar water splitting. *J. Mater. Chem. A* 3, 2485-2534 (2015).

21. Janczarek, M., Wei, Z., Endo, M. Ohtani, B. & Kowalska, E. Silver- and copper-modified decahedral anatase titania particles as visible light-responsive plasmonic photocatalyst. *J. Photonics Energy* **7**, 012008 (2017).

22. Guo, J. *et al.* Boosting hot electrons in hetero-superstructures for plasmon-enhanced catalysis. *J. Am. Chem. Soc.* **139**, 17964-17972 (2017).
